# Nipah Virus Outbreak in Kerala State, India Amidst of COVID-19 Pandemic

**DOI:** 10.3389/fpubh.2022.818545

**Published:** 2022-02-17

**Authors:** Pragya D. Yadav, Rima R. Sahay, Anukumar Balakrishnan, Sreelekshmy Mohandas, Chandni Radhakrishnan, Mangesh D. Gokhale, R. Balasubramanian, Priya Abraham, Nivedita Gupta, A. P. Sugunan, Rajan Khobragade, Kalpana George, Anita Shete, Savita Patil, Ullas Padinjaremattathil Thankappan, Hitesh Dighe, Jijo Koshy, Vivek Vijay, R. Gayathri, P. Jayesh Kumar, Asma Rahim, A. Naveen, Sarala Nair, V. R. Rajendran, V. Jayasree, Triparna Majumdar, Rajlaxmi Jain, Prasanth Viswanathan, Deepak Y. Patil, Abhinendra Kumar, Dimpal A. Nyayanit, Prasad Sarkale, Ashwini Waghmare, Shrikant Baradkar, Pranita Gawande, Poonam Bodke, Kaumudi Kalele, Jyoti Yemul, Sachin Dhaigude, Manjunath Holepannawar, Sanjay Gopale, Ganesh Chopade, Shilpa Ray, Priyanka Waghmare, Jitendra Narayan, Basavaraj Mathapati, Manoj Kadam, Abhimanyu Kumar, Annasaheb Suryawanshi, Beena Philomina Jose, Saritha Sivadas, N. P. Akash, T. V. Vimisha, K. V. Keerthi

**Affiliations:** ^1^Indian Council of Medical Research-National Institute of Virology, Pune, India; ^2^Indian Council of Medical Research-National Institute of Virology, Kerala Unit, Alappuzha, India; ^3^Department of Medicine, Government Medical College, Kozhikode, India; ^4^Epidemiology and Communicable Diseases Division, Indian Council of Medical Research, New Delhi, India; ^5^Health and Family Welfare Department, Government of Kerala, Thiruvananthapuram, India; ^6^Department of Microbiology, Government Medical College, Kozhikode, India; ^7^Department of Community Medicine, Government Medical College, Kozhikode, India; ^8^National Health Mission, Kozhikode, India; ^9^Health Department, Kozhikode, India; ^10^Government Medical College, Kozhikode, India; ^11^District Medical Office of Health, Health Department, Kozhikode, India

**Keywords:** Nipah virus (NiV), Kerala, *Pteropus medius*, bats, seropositivity

## Abstract

We report here a Nipah virus (NiV) outbreak in Kozhikode district of Kerala state, India, which had caused fatal encephalitis in a 12-year-old boy and the outbreak response, which led to the successful containment of the disease and the related investigations. Quantitative real-time reverse transcription (RT)-PCR, ELISA-based antibody detection, and whole genome sequencing (WGS) were performed to confirm the NiV infection. Contacts of the index case were traced and isolated based on risk categorization. Bats from the areas near the epicenter of the outbreak were sampled for throat swabs, rectal swabs, and blood samples for NiV screening by real-time RT-PCR and anti-NiV bat immunoglobulin G (IgG) ELISA. A plaque reduction neutralization test was performed for the detection of neutralizing antibodies. Nipah viral RNA could be detected from blood, bronchial wash, endotracheal (ET) secretion, and cerebrospinal fluid (CSF) and anti-NiV immunoglobulin M (IgM) antibodies from the serum sample of the index case. Rapid establishment of an onsite NiV diagnostic facility and contact tracing helped in quick containment of the outbreak. NiV sequences retrieved from the clinical specimen of the index case formed a sub-cluster with the earlier reported Nipah I genotype sequences from India with more than 95% similarity. Anti-NiV IgG positivity could be detected in 21% of *Pteropus medius* (*P. medius*) and 37.73% of *Rousettus leschenaultia* (*R. leschenaultia*). Neutralizing antibodies against NiV could be detected in *P. medius*. Stringent surveillance and awareness campaigns need to be implemented in the area to reduce human-bat interactions and minimize spillover events, which can lead to sporadic outbreaks of NiV.

## Introduction

Nipah virus (NiV) causes a highly lethal disease with acute severe encephalitis and acute respiratory distress syndrome in humans. The disease has been enlisted as a priority disease in the Research and Development blueprint of the WHO from the year 2015. NiV is a *Paramyxovirus* that was identified for the first time during an outbreak of severe encephalitis among the pig farmers in Malaysia in 1998 (1). The virus is transmitted to humans by direct contact with the respiratory secretions or body fluids of infected animals, such as bats and pigs, or by consumption of contaminated fruits/palm sap. Both animal-to-human and human-to-human transmission have been documented (1–3).

Subsequently, an outbreak of encephalitis among the human population was observed in Meherpur, Bangladesh in 2001 where the source of infection was traced down to drinking the contaminated raw palm sap or climbing the trees coated with bat excrement (4). India has witnessed two outbreaks of NiV encephalitis in the eastern state of West Bengal, bordering Bangladesh (5, 6). A case fatality of 70–100% was observed during these two outbreaks (5, 6). Since 2010, the Indian Council of Medical Research-National Institute of Virology (ICMR-NIV), Pune has taken up the surveillance of NiV in bat populations across the country. During this, the presence of NiV was detected among *Pteropus medius* (*P. medius*) from Maynaguri, West Bengal in 2010 and Cooch Bihar district, West Bengal, and Dhubri district, Assam in 2015 (7, 8).

After a decade of the last outbreak, a dreadful emergence of NiV was observed in Kozhikode district, Kerala State during May 2018 with a case fatality rate of 89% (9). The outbreak was contained with the quick actions of the national and state health systems (10). A year later, another outbreak was reported from Ernakulam district, Kerala with a single case. Due to the early detection; the further spread of the virus was quickly curtailed. A detailed outbreak investigation to find the source of NiV infection was carried out by ICMR-NIV, Pune during these outbreaks, which showed the presence of NiV and anti-NiV antibodies in *P. medius* (11). Recently a NiV outbreak was reported in Kozhikode Kerala in September 2021, where a 12-year-old male who presented with acute encephalitis and tested positive for NiV, succumbed to the infection.

Here, we describe the NiV outbreak management in Kozhikode district, Kerala, India with emphasis on the field laboratory setup and quick diagnosis along with the bat survey to trace the source of infection. Immediately after confirming the NiV infection on 4 September 2021, the outbreak containment response was initiated in the state of Kerala.

## Materials and Methods

### Case History

**Figure 1:**
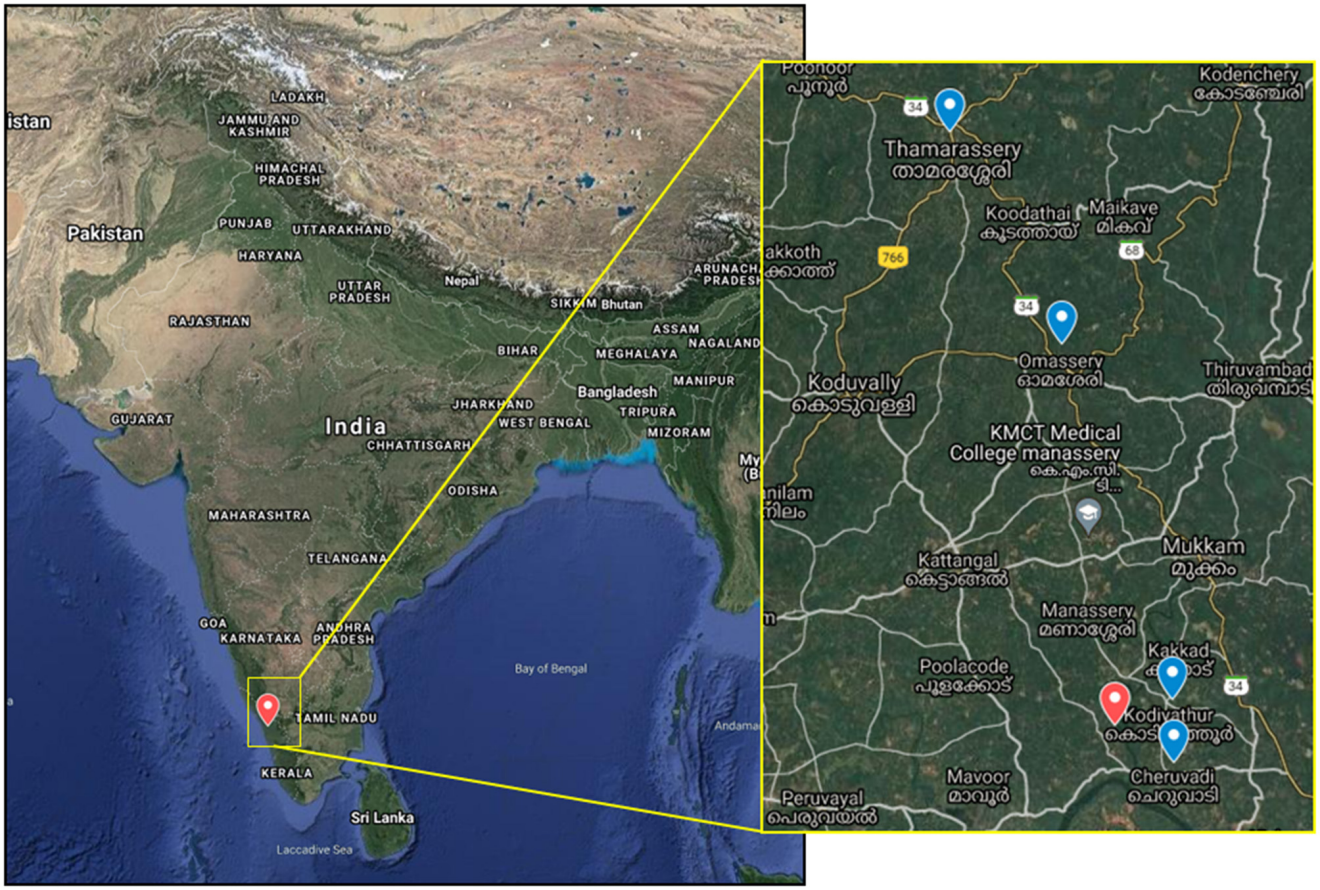
The map shows the Nipah virus outbreak location in India and in the inset, the location of the index case house (red icon), and the four bat sampling sites (blue icon).

On 29 August 2021, a 12-year-old boy (index case) resident of Pazhoor ward of Chathamangalam Panchayat, Kozhikode district, Kerala state, India developed low-grade fever (Figure 1). The family of the boy took him to a nearby private clinic (Hospital-1) and sought treatment for fever. On 31 August 2021, the condition of the boy deteriorated and boy was transferred to another hospital (Hospital-2). On 1 September 2021, the patient's condition deteriorated further and the patient developed symptoms of acute encephalitis and myocarditis. The symptoms were headache followed by disorientation and lack of consciousness. Signs of myoclonus and autonomic dysfunction were also observed. On the request of the family, the patient was transferred to a tertiary care hospital in Kozhikode (Hospital-3). MRI of the brain showed multiple small infarcts in the cerebellum, cerebrum, medulla oblongata, and pons. With a high suspicion of NiV infection, clinical samples of the patients, such as plasma, ethylenediaminetetraacetic acid (EDTA) blood, serum, and cerebrospinal fluid (CSF) samples, endotracheal (ET) secretion, and bronchial wash were sent to ICMR-NIV on 3 September 2021.

### Bat Trapping and Sample Collection

For understanding the source of NiV infection and considering a brief history given by the father of the index case regarding the consumption of fruit from the orchard near the house, bat sampling was done during the period of 11–18 September, 2021. The sampling was performed with the prior approval from the Institutional Animal Ethics Committee, Institutional Biosafety Committee of ICMR-NIV, Pune, and the Principal Chief Conservator of Forests, Government of Kerala. Four roosting sites were chosen from the nearby vicinity of the index case house for sample collection (Figure 1). These sites were in Kodiyathur (1 km from index case house), Cheruvadi (4 km), Omassery (12 km), and Thamarassery (18 km). Bats were trapped using the mist nets as described earlier [11]. Body weight, sex, secondary sexual characters, and forearm length were noted. Blood (*n* = 91), throat (*n* = 102), and rectal swab (*n* = 102) samples were collected from the trapped bats following isoflurane anesthesia. Species identification was performed by mitochondrial Cytochrome b gene PCR as described earlier (12).

### Real-Time Quantitative Reverse Transcription (qRT)-PCR

Real-time PCR (qRT-PCR) was performed on the samples for the NiV diagnosis as described earlier (13). Two hundred microliter of serum/swab samples were used for RNA extraction in an extraction machine using Magmax Viral RNA isolation kit as per the instructions of the manufacturer. For severe acute respiratory syndrome coronavirus 2 (SARS-CoV-2) detection, qRT-PCR was performed for throat/nasal swab samples using primers for E gene as described earlier (14).

### Anti-Nipah Human Immunoglobulin M (IgM) and Immunoglobulin G (IgG) ELISA

The assays were performed as described earlier (11).

### Whole Genome Sequencing (WGS)

To identify the NiV genotype, WGS was carried out on different clinical samples obtained from the patient. RNA was extracted from the clinical samples, and WGS was performed using the methods described earlier (15). The viral reads generated were analyzed using reference-based mapping, performed in CLC Genomics Workbench version 21.0.4. In order to retrieve the complete genome sequence of the virus, all the generated reads were mapped to the reference genome. Phylogenetic analyses and the amino acid variations of the retrieved NiV sequence with the other representative NiV sequences of earlier outbreaks were performed. SARS-CoV-2 positive samples were sequenced as per the method described earlier to identify the lineage in circulation (16).

### Anti-NiV bat IgG ELISA

The assay was performed as described earlier (15).

### Plaque Reduction Neutralization Test

Heat inactivated bat serum samples were mixed with NiV (GenBank accession number: MH523642) containing 50 plaque-forming units in a 1:1 ratio so as to make a final 10-fold dilution of the serum virus mixture. Anti-Nipah IgG positive mice serum was used as positive control, and anti-Nipah IgG negative mice serum was used as a negative control. The mixture was incubated for 1 h and was added to a 24-well tissue culture plate containing a confluent monolayer of Vero CCL-81 cells. The plate was incubated in a CO_**2**_ incubator at 37°C for 1 h and an overlay medium containing 2% **c**arboxymethyl cellulose in 2 × Minimal Essential Media with 2% fetal bovine serum was added after removing the inoculum. The plate was further incubated at 37°C in a CO_2_ incubator for 3 days. After removing the overlay medium, the plate was washed and stained with amido black. The plaques were counted. The titer was defined as the highest serum dilution that resulted in a 50% (PRNT50) reduction in the number of plaques.

## Results

### Detection and Confirmation of NiV Infection

Nipah virus infection was confirmed by detection of viral RNA in the blood (7 × 10^5^ genome copies/ml), bronchial wash (3.5 × 10^4^ genome copies/ml), ET secretion (1.1 × 10^7^ genome copies/ml), and CSF (3.5 × 10^4^ genome copies/ml) by qRT-PCR and by the detection of anti-Nipah IgM antibodies in serum sample of the patient. Virus isolation attempts from the samples in Vero CCL-81 cells were not successful. Other etiological agents, such as Japanese Encephalitis, West Nile virus, Dengue, Chikungunya, Influenza A and B, Respiratory Syncytial Virus A and B, Parainfluenza 1–4, human metapneumovirus, Rhinovirus Adenovirus, Zika, were also ruled out simultaneously.

On 4 September 2021, the NiV outbreak in Kerala was declared by the Ministry of Health and Family Welfare, Government of India. The patient succumbed to the infection on 5 September 2021. Contact tracing and isolation of high-risk contacts, augmentation of laboratory testing capacity, bat sampling, and laboratory investigations were undertaken following the event as described below.

### Establishment of an On-site Field NiV Diagnostic Facility

A team from ICMR-NIV Pune had setup a field diagnostic laboratory in the Department of Microbiology, Government Medical College, Kozhikode, Kerala by 6 September 2021 following all the essential biosafety guidelines and standard operating procedures.

### Risk Categorization and Contact Tracing

After the declaration of the NiV outbreak, systematic field investigations were undertaken to identify the epidemiologically linked close contacts, such as healthcare workers, family members, neighbors, and bystanders. The close contacts were classified into primary contacts and secondary contacts and were further grouped into high-risk and low-risk contacts. The high-risk category included individuals with either a history of direct contact with body fluids (blood, urine, saliva, vomitus, etc.) of the confirmed NiV case or a probable case that was died without laboratory confirmation or having spent about 12 h nearby or in closed space with confirmed NiV case. The low-risk contacts were categorized as those having contact with the confirmed NiV case through touching or contact with clothes, linen, or any other items.

**Figure 2:**
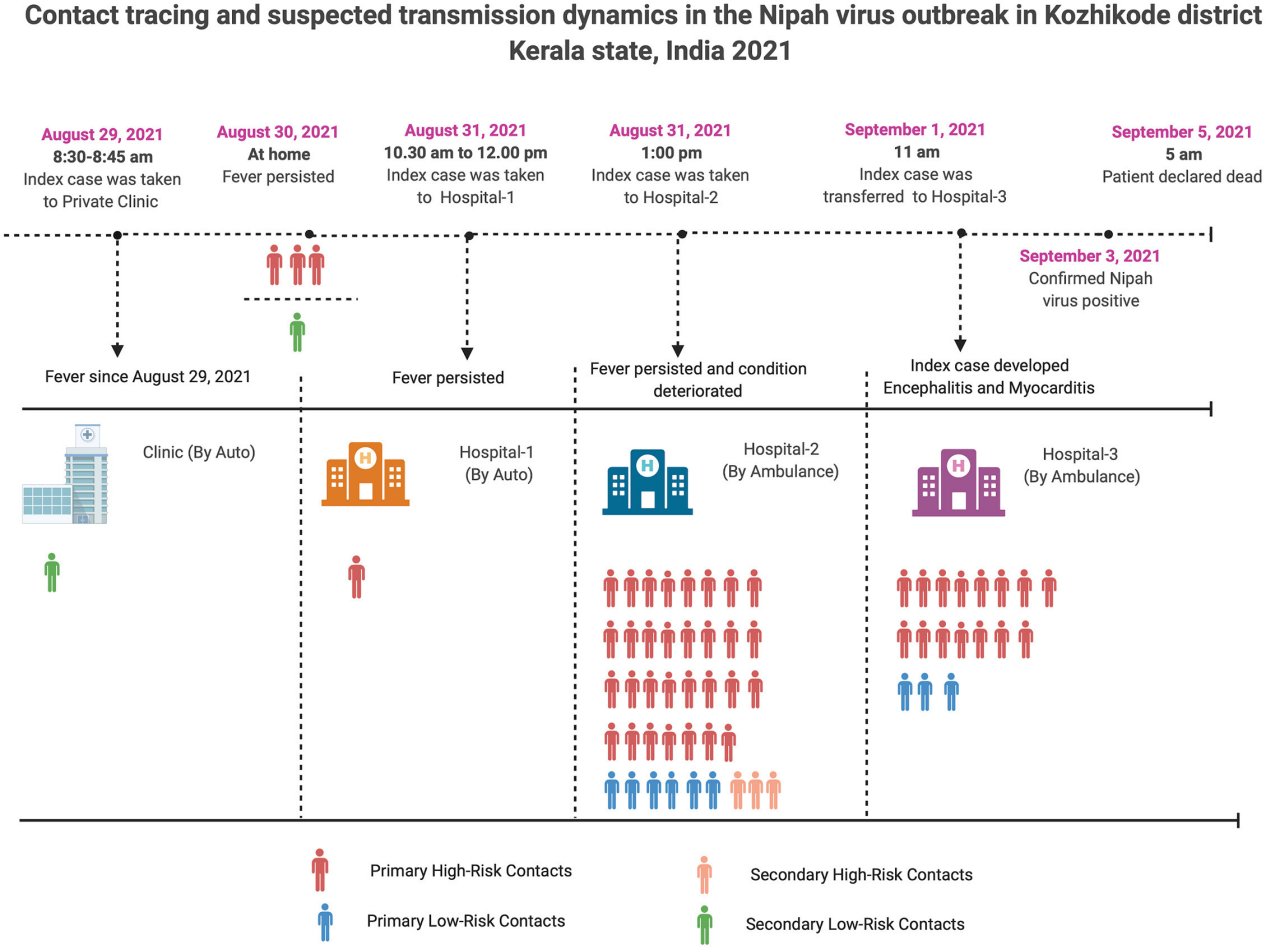
Contact tracing and probable transmission dynamics in the Nipah virus outbreak in Kozhikode district, Kerala state, India, 2021.

A total of 240 contacts were listed and among them 64 close contacts [33 women/31 men] were identified and grouped into primary high-risk (*n* = 50) and low-risk (*n* = 9) contacts; secondary high-risk (*n* = 3) and low-risk (*n* = 2) contacts (Figure 2). Out of 59 primary contacts, 40 were asymptomatic while all the 5 secondary contacts were asymptomatic (Table S1). Samples of the symptomatic contacts (*n* = 19) were shipped to the Biosafety Level 4 (BSL-4) laboratory of ICMR-NIV, Pune for diagnosis. The field laboratory was utilized for testing the 45 asymptomatic contacts and 61 non-epidemiologically linked suspected NiV cases (cases from nearby districts of Kozhikode district with Acute Encephalitis Syndrome manifestations). All the close contacts and non-epidemiologically linked suspected cases were found negative for NiV by qRT-PCR and ELISA.

Considering the ongoing Coronavirus Disease-19 (COVID-19) pandemic, all the close contacts were also screened for SARS-CoV-2. The throat/nasal swab of the 12 close contacts (symptomatic-8 and asymptomatic-4) was found positive for SARS-CoV-2 by qRT-PCR (Table S1). On sequencing of SARS-CoV-2 positive samples (*n* = 12), Delta variant (B.1.167.2) and its derivatives (AY.26) were detected in 10 and two cases, respectively.

### Genomic Characterization of NiV From Clinical Specimens of the Index Case

**Figure 3:**
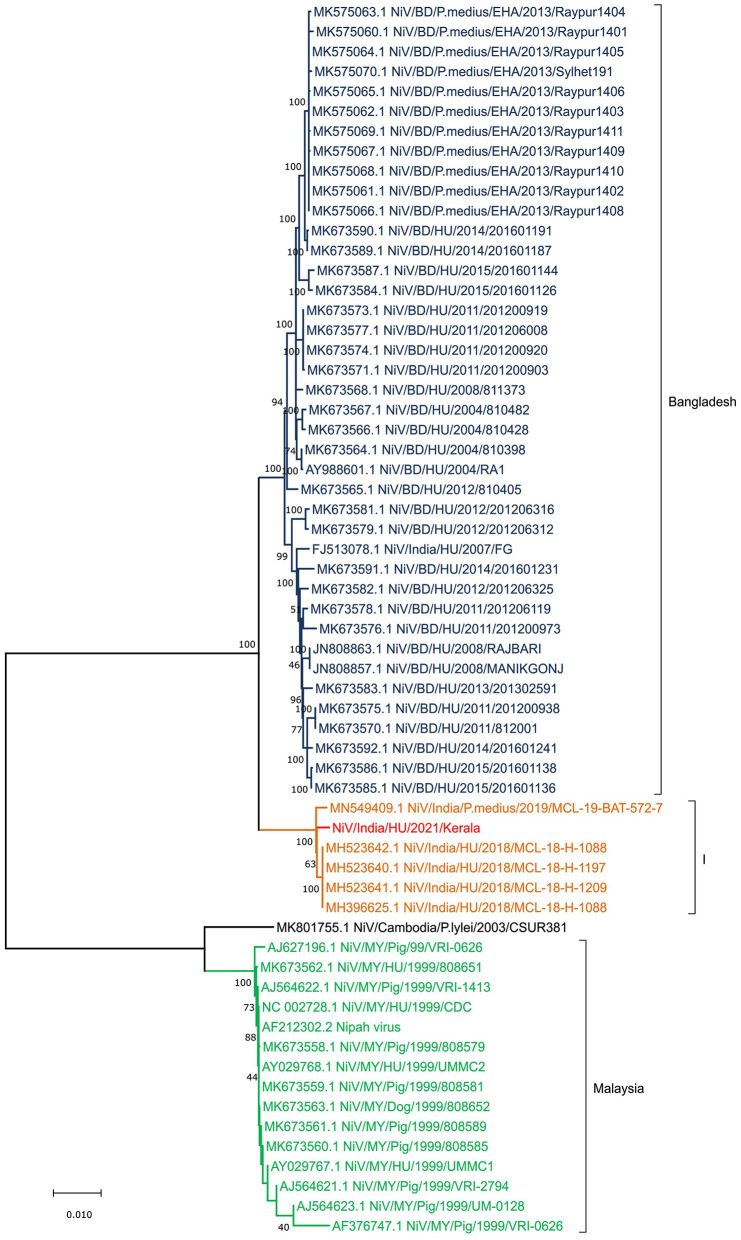
Neighbor Joining tree of Nipah virus full genome obtained from the samples of the index case in Kerala outbreak 2021.

A phylogenetic analysis was performed for the retrieved NiV sequence (17,066 nucleotides) with the other representative NiV sequences of the earlier outbreaks (Figure 3; Table S2). The retrieved sequence is clearly segregated from the Bangladesh NiV sequences and clustered into earlier described Indian (“I”) genotype. The retrieved sequence showed 99.62 and 99.51% nucleotide similarity (PNS) with sequences obtained from human samples during the 2018 NiV outbreak and *P. medius* samples during the 2019 outbreak respectively.

The different genes of NiV showed amino acid variation between the Bangladesh and I genotype clusters. The changes observed were in the N gene (S503N, P520S, E752G, Q758E, R818H, I820L, T919N, Q982R, A1162T, and G1216D), F gene (I15L), and G gene (R344M, I384V, V427I) of I genotype of the NiV compared to the NC_002728.1. Similarly, the changes were observed in the N gene (R505K, S900G, and D921N), G gene (R344K, K386E, and T498K), and L gene (R1262K and N 237D) when the Bangladesh (BD) NiV sequences were compared to the NC_002728.1 (Table 1).

**Table 1:** Amino acid changes in the different genotypes across representative human and bat sequences for genes encoded by NiV relative to NC 002728.1.

**Nipah virus strain**	**Genotype**	**Species**	**N gene**																									
			**503**	**505**	**520**	**594**	**597**	**614**	**752**	**758**	**761**	**818**	**820**	**830**	**831**	**833**	**843**	**844**	**845**	**900**	**919**	**921**	**954**	**958**	**982**	**1162**	**1188**	**1216**
NC 002728.1 NiV/MY/HU/1999/CDC Reference genome of Nipah virus	M	Human	**S**	**R**	**P**	**G**	**S**	**T**	**E**	**Q**	**R**	**R**	**I**	**Q**	**A**	**D**	**G**	**P**	**K**	**S**	**T**	**D**	**P**	**S**	**Q**	**A**	**M**	**G**
NiV/India/HU/2021/sample	I	Human	N	–	S	–	–	–	G	E	–	H	L	–	I	G	–	L	–	–	N	–	L	–	R	T	–	D
MH523642.1 NiV/India/HU/2018/MCL-18-H-1088	I	Human	N	–	S	–	–	–	G	E	–	H	L	K	I	G	–	L	–	–	N	–	L	N	R	T	–	D
MN549409.1 NiV/India/P.medius/2019/MCL-19-BAT-572-7	I	Bat	N	–	S	–	–	–	G	E	–	H	L	–	I	G	–	L	–	–	N	–	–	–	R	T	–	D
MK673592.1 NiV/BD/HU/2014/201601241	B	Human	–	K	–	–	P	–	–	–	K	–	–	–	I	G	–	–	–	G	–	N	–	–	–	–	–	–
MK575070.1 NiV/BD/P.medius/EHA/2013/Sylhet191	B	Bat	–	K	–	R	–	A	–	–	–	H	–	–	I	–	–	–	R	G	–	N	–	–	–	–	V	–
			**F gene**						**G gene**					**L gene**														
			**15**	**19**	**344**	**384**	**386**	**427**	**498**	**94**	**112**	**632**	**633**	**639**	**642**	**658**	**665**	**1262**	**2037**									
**NC 002728.1 NiV/MY/HU/1999/CDC**	**M**	**Human**	**I**	**M**	**R**	**I**	**K**	**V**	**T**	**I**	**K**	**N**	**V**	**N**	**N**	**H**	**T**	**R**	**N**									
NiV/India/HU/2021/sample	I	Human	L	–	M	V	–	I	–	T	–	–	–	–	C	Y	–	–	–									
MH523642.1 NiV/India/HU/2018/MCL-18-H-1088	I	Human	L	–	M	V	–	I	–	T	–	–	–	–	Y	Y	–	–	–									
MN549409.1 NiV/India/P.medius/2019/MCL-19-BAT-572-7	I	Bat	L	–	M	V	–	I	–	T	–	–	–	–	Y	–	–	–	–									
MK673592.1 NiV/BD/HU/2014/201601241	B	Human	–	I	K	–	E	–	K	–	–	–	–	–	Y	Y	–	K	D									
MK575070.1 NiV/BD/P.medius/EHA/2013/Sylhet191	B	Bat	–	–	K	–	E	–	K	T	R	S	M	D	–	Y	I	K	D									

*The bold values are the amino acid at positions mentioned in the table for each gene of the reference Nipah virus strain, NC 002728.1 NiV/MY/HU/1999/CDC*.

### Detection of Anti-NiV IgG Antibodies in bat Samples

The bat species sampled in the study included *P. medius [n* = *38 (juveniles* = *12, adults* = *26)], Rousettus leschenaultia [R. leschenaultia; n* = *63 (juveniles* = *19, adults* = *44)]*, and *Pipistrellus sp. (n* = *1)* (Table S3). All the bat samples were found to be negative for Nipah viral RNA. The serum samples of *P. medius [n* = *8 (juveniles* = *2, adults* = *6)]*, and *R. leschenaultia [n* = *20 (juveniles* = *7, adults* = *13)]* were tested positive for anti-NiV IgG antibodies. The *P. medius* samples from two sites, i.e., Kodiyathur and Thamarassery, showed positivity of 20 and 56% by ELISA, respectively, and were further confirmed by the plaque reduction neutralization test (PRNT). Two samples were excluded from the assay due to insufficient sample quantity. *R. leschenaulti* samples, which showed seropositivity (1/4 bats from Kodiyathur and 19/38 bats from Cheruvadi) by ELISA was found negative for neutralizing antibodies.

## Discussion

Nipah virus outbreaks have been reported from Malaysia, Singapore, Bangladesh, and India with a range of clinical presentations and case fatality rates of 40–100% (1–6, 8). Malaysia and Singapore had reported a single NiV outbreak episode whereas Bangladesh reports annual outbreaks of NiV (4). In India, NiV outbreaks have been localized to two regions, i.e., in the Northeastern state of West Bengal and in the southernmost state of Kerala, which are separated by a distance of more than 2,000 km (5, 6, 9–11). NiV outbreaks are mostly sporadic and the magnitude of the outbreak can be restricted by prompt public health response and containment measures. In 2018, the first NiV outbreak was reported in Kerala state with a significant number of losses of lives (91% fatality) and in the years 2019 and 2021 outbreaks in Kerala reported only a single case without any further human to human transmission (9, 11). The diagnosis of the NiV infection is difficult to consider the overlapping presentations of acute respiratory infections and encephalitis syndromes. In the current SARS-CoV-2 pandemic scenario, the diagnosis became even more challenging considering the overlapping clinical features. The quick outbreak containment response and the biosafety practices followed during the COVID-19 pandemic period in Kerala state might have helped in preventing further transmission and restricting the outbreak to a single case.

The role of intermediate hosts, such as pigs and bats, has been demonstrated in the NiV transmission cycle in the previously reported outbreaks (1, 3, 4). *Pteropus* genus of bats appears to be the major reservoir of the NiV (8, 17). *P. medius* is the only *Pteropus* genus bat present in the Indian subcontinent (18). Pteropus bats have shown NiV RNA positivity or seropositivity from Indian states, such as West Bengal, Assam, Haryana, and Kerala, indicating the risk of spillover (7, 8, 11, 19). Viral genome recovered from the current outbreak also clustered with the previously reported NiV genotype from Kerala in bats and human samples. This suggests a stable genotype that circulates locally in the bat population in Kerala. Research has shown that NiV evolves at a slower rate compared to other similar RNA viruses (20, 21). In 2018, the outbreak occurred in Perambra, Kozhikode, which is about 40 km far from the location of the present outbreak (9). A total of 23 cases and 21 deaths were reported. In total, 21% of the *P. medius* bats surveyed in the vicinity of the index case residence were found positive for NiV. In 2019, NiV RNA positivity was documented in *P. medius* bats from Thodupuzha, which is more than 200 km from Kozhikode district (11) *Pteropus* species movement could also play an important role in virus spread, but in India, such records are not available. Home ranges of *P. medius* appear to be smaller than *Pteropus vampyrus* (*P. vampyrus*), another frugivorous bat in Malaysia and this depends on food availability (17). Studies on the *Pteropus* species movement and connectivity among the bat populations could help us in understanding the potential of virus spread to bat colonies of adjacent areas.

Nipah virus RNA could not be detected in any of the bat samples in the present study. Previous studies have also reported a low PCR positivity in *P. medius* (20–22). Virus shedding in bats is driven by multiple factors, such as individual immune status, pregnancy, virus recrudescence, and stress (21, 22). Re-infection in adult bats is possible in approximately 7 years (22). We detected 25 and 3.2% NiV positivity in bats from outbreak regions of Kerala in 2018 and 2019 (9, 11). High seroprevalence in the bat colonies can dampen virus transmission (17). The seasonality of NiV outbreaks has been reported linked to the breeding season and fruit harvesting season (23). Unlike the previously reported outbreaks in the month of May and June in Kerala, the present outbreak was in August end. A cross-sectional spatial study conducted between 2006 and 2012 in Bangladesh reports the ability of NiV shedding by *P. medius* throughout the year (17).

During the current outbreak investigations, we could detect antibodies in a total of 21% *P. medius* bats. If we further classify this positivity, site wise, a 20% positivity was observed in Kodiyathur, a site within 1 km distance from the index case house and 55.6% in Thamarassery, a site 18 km far from this outbreak. During the investigation, 2 juvenile *P. medius* bats were found positive for anti-NiV IgG antibodies. The seroprevalence in the juveniles is an indicator of the enzootic cycle of NiV in the recent past. The waning of immunity with time and loss of maternal antibodies could affect the transmission dynamics within the bat colonies (21, 22). Local NiV epizootics in bats contribute to the outbreaks in Bangladesh (17). Sporadic natures of outbreaks in the case of NiV are explained by the NiV dynamics in the resident bat colonies and the bat-human interface resulting in spillover. Even though antibodies could be detected in bats from the outbreak area, viral RNA could not be detected in the bats. Hence we could not directly link the source of infection to bats.

Non-pteropid bats have shown NiV seropositivity in many countries (24–27). In India, other than *P. medius*, bats, such as *R. leschenaulti*a and *Pipistrellus* (*P. pipistrellu*s), have shown seropositivity by ELISA (25). Interspecies transmission could be possible with other frugivorous bats as they share the same habitat for food resources. Similarly, in the present study, we could detect seropositivity in *R. leschenaulti*a bats by ELISA. But the absence of NAb against NiV in these bats' points to the possibility of detection of related Henipaviruses in these bats. Similar observations are reported in Vietnam where the ELISA positive samples of *R. leschenaulti*a failed to show any NAb, indicating the probability of cross-reacting antibodies against non-neutralizing epitopes (26). Antibody positivity by ELISA and Western Blot in *R. leschenaulti*a has been reported from China, but neutralization studies were not performed in the samples (27).

The findings from the current outbreak suggest that spillover of NiV infection in humans is sporadic and the seroprevalence in bats indicates the prevalence of the NiV infections in the *P. medius* population. Stringent surveillance measures and awareness campaigns to limit interactions with bats need to be implemented in the area as the NiV transmission dynamics depends on multiple host factors that includes the human behavior and human-bat interface. For early detection and containment of NiV outbreaks, it is critical to strengthen human surveillance for Acute Encephalitis Syndrome and Severe Acute Respiratory Infection, including testing for NiV in susceptible areas.

## Data Availability Statement

The data presented in the study are deposited in the GenBank and the accession numbers are OM135495 for Nipah virus, OK428833 to OK428845, OK490612 and OK490613 for Cytochrome B sequences. All the other data are available in the article/Supplementary Material.

## Ethics Statement

The studies involving human participants were reviewed and approved by Institutional Ethics Committee. The patients/participants provided their written informed consent to participate in this study. The animal study was reviewed and approved by ICMR-National Institute of Virology, Institutional Animal Ethics Committtee.

## Author Contributions

PY, APS, PA, NG, CR, RK, NA, and VR supervised and co-ordinated the outbreak response. RS, AB, APS, SM, KG, and BJ established the field laboratory. MG and RB performed the bat survey site selection and supervision of the bat trapping and sample collection. PY, RS, SM, UT, ASh, and DP supervised the laboratory investigations. CR, RG, PK, and AR were involved in close contact isolation and sampling. SP, HD, JK, VV, PV, SS, AN, VV, and KVK performed the field laboratory sample processing and testing. NA, SN, and VJ were involved in contact tracing and co-ordination of outbreak response. RJ, PB, SR, and PW performed ELISA. DN, AbhinK, and TM performed the next-generation sequencing and analysis. PS and SB performed the neutralization assay. AW, PG, KK, and JY performed the laboratory sample processing for PCR, sequencing, and testing. SD, MH, SG, and GC performed the bat trapping and sample collection on-site. NG and JN supported the outbreak response management. SM, BM, AbhimK, MK, SD, and ASu were involved in laboratory bat sample processing. All authors contributed to the article and approved the submitted version.

## Funding

ICMR supported the funding for this study under the project ‘Countrywide survey of Nipah virus in Pteropus bats'. The funders had no role in study design, data collection or interpretation, or the decision to submit the work for publication. The findings and conclusions in this study are of the authors.

## Conflict of Interest

The authors declare that the research was conducted in the absence of any commercial or financial relationships that could be construed as a potential conflict of interest. The reviewer PB declared a shared affiliation with several of the authors, PY, RS, AB, SM, MG, RB, PA, NG, APS, ASh, SP, UT, HD, JK, VV, TM, RJ, DP, AbhinK, DN, PS, AW, SB, PG, PB, KK, JY, SD, MH, SG, GC, SR, PW, JN, BM, MK, AbhimK, and ASu, to the handling editor at the time of review.

## Publisher's Note

All claims expressed in this article are solely those of the authors and do not necessarily represent those of their affiliated organizations, or those of the publisher, the editors and the reviewers. Any product that may be evaluated in this article, or claim that may be made by its manufacturer, is not guaranteed or endorsed by the publisher.
